# Microbial succession accompanies increased antibiotic resistance risk during grass carp (*Ctenopharyngodon idella*) spoilage under ambient household conditions

**DOI:** 10.1186/s12866-026-04924-w

**Published:** 2026-03-09

**Authors:** Zehan Shen, Zuowu Zhang, Jingrong Gao, Jiao Chen, Qiuxiang Xu, Dongyi Li, Li Zeng, Dengmiao Cheng, Kai Wang, Jiayu Zhang, Jonathan W.C. Wong

**Affiliations:** 1 Research Center for Eco-environmental Engineering, Dongguan University of Technology, Dongguan, 523808 China; 2 School of Food and Pharmacy, Zhejiang Ocean University, Zhejiang, 316022 China

**Keywords:** Fish spoilage, Grass carp, Microbial community, Specific spoilage organism, Antibiotic resistance gene

## Abstract

Understanding fish spoilage mechanisms under household storage conditions is critical for food safety in regions with limited cold chain infrastructure, where ambient storage remains common practice. This study investigated the spoilage dynamics, microbial succession, and antibiotic resistance gene (ARG) proliferation in grass carp stored under simulated household conditions at 13.0 ± 3.4 °C using three packaging scenarios. The biogenic amine index (BAI) of fish exceeded 50 mg/kg within 16 h, marking early spoilage onset. After 64 h, K-values surpassed 60%, TVB-N exceeded the safety limit of 20 mg/100 g, and BAI reached over 220 mg/kg, indicating advanced spoilage. 16S rRNA amplicon sequencing demonstrated dramatic microbial community shifts from Cyanobacteriota-dominated fresh samples to Pseudomonadota-dominated spoilage communities, with *Aeromonas* emerging as the primary specific spoilage organism (SSO), increasing from 0.001% to 67.2% at 64 h. Pathogen abundance escalated from 0.06% to 72.2% in muscle tissues, posing substantial food safety risks. Distinct microbial community structures were observed across tissue types (muscle vs. gut) and packaging treatments, with storage time exerting the strongest selective pressure on community composition. Metagenomic analysis revealed progressive ARG enrichment, with surface samples exhibiting 2.6-fold higher total ARG abundance and 3.8-fold greater ARG type richness compared to the fresh gut baseline by 24 ~ 64 h. Rapid ARG enrichment was detected during early spoilage (24 h), representing a critical food safety concern. Notably, carbapenem resistance genes (e.g., OXA-12, *cphA6*) were substantially enriched, underscoring the high risk posed by these clinically relevant resistance genes. These findings demonstrate that grass carp stored under ambient household conditions maintain acceptable quality for < 16 h, necessitating immediate consumption or cold chain implementation to ensure food safety and minimize ARG dissemination.

**Supplementary Information:**

The online version contains supplementary material available at 10.1186/s12866-026-04924-w.

## Introduction

Grass carp (*Ctenopharyngodon idellus*), a traditional freshwater aquaculture species in China, represents the world’s most commercially significant farmed fish by production volume [1]. According to The State of World Fisheries and Aquaculture 2024, grass carp currently ranks as the highest-yielding fish species globally, with annual production exceeding 6 million metric tons, of which China contributes 5.94 million metric tons [2]. However, the post-harvest perishability of grass carp presents formidable challenges to quality maintenance and food safety assurance, particularly in distribution channels lacking adequate cold chain infrastructure [3]. In contemporary market systems, grass carp is often transported over short distances and retailed in basic packaging, such as polyethylene (PE) bags or unsealed plastic crates without any temperature control [4]. This packaging paradigm, while economically expedient for small-scale vendors, wet markets, and peri-urban distribution networks, creates optimal conditions for rapid microbial proliferation and biochemical deterioration. Unlike industrialized cold chain systems employing modified atmosphere packaging, vacuum sealing, or cryogenic preservation, conventional ambient storage exposes fish tissues to warm, humid microenvironments (typically 10 ~ 20 °C) that accelerate both exogenous microbial invasion and endogenous autolytic cascades [5].

Fish spoilage constitutes a multifactorial process governed by two synergistic pathways. Microbial-mediated deterioration is driven by specific spoilage organisms (SSOs), predominantly *Pseudomonas*, *Shewanella*, and *Aeromonas*, that rapidly colonize epithelial surfaces and muscle tissues following harvest mortality [6, 7]. These SSOs catabolize proteins, free amino acids, and trimethylamine oxide (TMAO), generating volatile basic nitrogen (TVB-N), biogenic amines, and sulfur compounds that collectively define the “spoilage bouquet” [8]. Concurrently, endogenous autolytic mechanisms proceed independently through residual enzymatic processes. ATP degradation follows a well-characterized cascade (ATP → ADP → AMP → IMP → HxR → Hx), with the K-value serving as the gold-standard freshness indicator [9]. Cathepsin-mediated proteolysis disrupts myofibrillar architecture, causing texture deterioration, while endogenous lipases catalyze oxidative rancidity [10]. High-throughput sequencing has demonstrated that spoilage progression involves deterministic community succession rather than stochastic bacterial growth, with environmental selective pressures shaping reproducible SSO emergence patterns [11].

Despite these advances, critical knowledge gaps persist. First, existing research disproportionately emphasizes low-temperature cold chain or modified atmosphere preservation scenarios, particularly neglecting the conventional ambient storage that prevails in resource-limited regions. Second, comparative analyses of spoilage dynamics across different household packaging methods under ambient conditions remain scarce, limiting practical guidance for small-scale vendors. Third, the tissue-specific distribution and succession patterns of spoilage and pathogenic microbes in muscle versus gut compartments during ambient storage are poorly characterized. Fourth, and most critically from a public health perspective, the proliferation and potential transfer of antibiotic resistance genes (ARGs) harbored by spoilage microbiota during fish storage constitute a grossly understudied dimension of food safety risk. The emergence of antimicrobial resistance as a global health crisis has intensified scrutiny of ARG dissemination pathways through food systems [12, 13]. Yet systematic characterization of ARG dynamics during fish spoilage, including temporal enrichment patterns and tissue-specific distribution, remains conspicuously absent from the literature.

To address these interconnected knowledge gaps, this study employed an integrated approach combining biochemical quality monitoring, microbial community profiling, and metagenomic ARG surveillance to systematically dissect grass carp spoilage under simulated household storage conditions. Three packaging scenarios representing common household practices were evaluated: sealed PE bags, unsealed PE bags with partial air exchange, and unpacked fish with full environmental exposure. Our specific objectives are threefold: (1) quantify spoilage progression differences across the three packaging treatments under ambient temperature using biochemical deterioration markers; (2) characterize microbial community succession in muscle and gut tissues during spoilage, with emphasis on specific spoilage organisms and pathogenic bacteria dynamics; and (3) profile ARG proliferation patterns across storage duration, packaging conditions, and tissue compartments, identifying clinically relevant resistance determinants. This investigation aims to generate actionable evidence for optimizing household preservation strategies in resource-limited settings and refining food safety risk assessments associated with post-harvest spoilage processes.

## Methods

### Acquisition and processing of grass carp

Ninety female grass carp aged approximately 12 months were used in this study. Their average body weight ranged from 1.3 to 1.6 kg. The fish were obtained in December from a pond-cultured aquaculture farm located in Zhongshan City, Guangdong Province, China. The fish were euthanized using the pithing method to ensure humane treatment. Freshly euthanized specimens were rapidly cooled on ice and transported under refrigerated conditions to the laboratory for subsequent processing and analysis. The processed fish were subsequently allocated into three experimental groups to evaluate the impact of different packaging conditions on microbial dynamics during ambient storage: Group A consisted of fish packed in PE bags sealed with twist ties, which may still retain some oxygen, though less than the bags used for types B and C; Group B comprised fish packed in PE bags without sealing to allow partial air exchange; and Group C included unpacked fish with full environmental exposure. All groups were placed in individual plastic containers at room temperature to prevent insect contamination and cross-contamination between samples. Sampling was conducted at 8-hour intervals over a 72-hour ambient storage period, with three fish sampled at each time point to provide triplicate biological replicates. At each sampling point, the fish surface, abdomen, fins, and tail were thoroughly swabbed using sterile throat swabs to capture microbes on the fish surface, and the swab tips were aseptically broken off into sterile 2 mL microcentrifuge tubes. Following fish surface sampling, the abdominal cavity was carefully opened under sterile conditions, and approximately 20 g of dorsal muscle tissue was excised and transferred into sterile sealed bags for subsequent analysis. Additionally, intestinal content was gently squeezed out and collected into sterile 2 mL microcentrifuge tubes. All collected samples, including swabs, muscle tissue, and intestinal content, were immediately frozen at − 20 °C and stored until analysis. All measurements were performed in triplicate to ensure analytical precision and reproducibility.

### TVB-N determination

TVB-N content was quantified using a Kjeldahl nitrogen analyzer GL 700 (GreenKerry Instrument Co., Ltd., China) following the standard distillation-titration method. Frozen dorsal muscle samples were thawed at 4 °C, and skin and bones were carefully removed to obtain pure muscle tissue. The muscle was then homogenized into a uniform paste using a sterile homogenizer. A 10 g aliquot of the homogenized sample was accurately weighed and transferred into a distillation tube, to which 75 mL of distilled water was added. After soaking for 30 min, 1 g of MgO was added to alkalize the solution and liberate volatile amines. The distillation tube was then immediately connected to the distillation unit and operated under manufacturer-specified preset conditions. The ammonia-containing distillate was titrated with standardized 0.1 mol/L HCl solution. The TVB-N content (expressed as mg N/100 g sample) was calculated based on the volume of HCl consumed during titration according to the formula described by Bekhit et al. [8].

### Biogenic amine analysis

Biogenic amines were quantified following the method described by Zhuang et al. [11] with minor modifications. Homogenized fish muscle tissue (5 g) was extracted with 10 mL of ice-cold 0.6 M perchloric acid and centrifuged at 10,000 g for 5 min at 4 °C. The supernatant was collected, and the precipitate was re-extracted under identical conditions to ensure complete amine recovery. The combined supernatants were adjusted to a final volume of 25 mL with 0.6 M perchloric acid. For derivatization, an aliquot of 0.4 mL extract was sequentially mixed with 80 µL of 2 M NaOH and 1200 µL of saturated sodium bicarbonate, followed by the addition of 0.8 mL of dansyl chloride solution (10 mg/mL in acetonitrile). The reaction mixture was incubated at 40 °C for 45 min in a water bath, then quenched with 40 µL of 25% (v/v) ammonia solution and allowed to stabilize at room temperature for 20 min. The derivatized sample was diluted to 2 mL with acetonitrile, filtered through a 0.22 μm membrane, and analyzed by high-performance liquid chromatography (HPLC) equipped with a C18 column (250 mm × 4.6 mm, 5 μm) (Thermo Fisher, USA) and ultraviolet detector (λ = 254 nm). Gradient elution was performed using methanol and 0.1 M ammonium acetate buffer containing 0.1% (v/v) formic acid as follows: 70:30 (v/v, 0 ~ 5 min), 60:40 (v/v, 5 ~ 15 min), and 30:70 (v/v, 15 ~ 45 min) at a flow rate of 0.8 mL/min.

### K-value determination

Homogenized fish muscle tissue (2 g) was extracted with 20 mL of 10% (v/v) perchloric acid solution, thoroughly mixed by vortexing, and centrifuged at 8,000 g for 10 min at 4 °C to remove precipitated proteins. The resulting precipitate was subjected to a second extraction with an equal volume of 5% (v/v) perchloric acid, followed by centrifugation. The two supernatants were pooled and carefully titrated with 1 M NaOH solution to adjust the pH to 6.2 ~ 6.3. The neutralized extract was then filtered through a 0.22 μm organic phase membrane for HPLC analysis. The HPLC system was equipped with a C18 column (250 mm × 4.6 mm, 5 μm) and an ultraviolet detector set at 254 nm. The HPLC separation was performed using an isocratic mobile phase consisting of 0.02 M KH_2_PO_4_ and 0.02 M K_2_HPO_4_ at a ratio of 50:50 (v/v), with a total runtime of 45 min per sample. To quantify freshness, the K value was calculated based on the concentrations of triphosphate (ATP) and its degradation products, which include adenosine diphosphate (ADP), adenosine monophosphate (AMP), inosine monophosphate (IMP), inosine (HxR), and hypoxanthine (Hx), using the standard formula: K value (%) = [(HxR + Hx) / (ATP + ADP + AMP + IMP + HxR + Hx)] × 100.

### Total sulfhydryl content determination

Total sulfhydryl content was quantified using a commercial assay kit (Nanjing Jiancheng Bioengineering Institute, China) according to the manufacturer’s protocol. Briefly, homogenized dorsal muscle tissue was mixed with the provided extraction buffer at the specified ratio, and the sulfhydryl groups were measured spectrophotometrically at 412 nm following reaction with 5,5’-dithiobis-2-nitrobenzoic acid.

### pH measurement

Muscle pH was determined using a calibrated pH meter. Homogenized fish muscle was mixed with sterile physiological saline (0.9% NaCl) at a ratio of 1:10 (w/v), vortexed vigorously for 30 s, and centrifuged at 8,000 g for 10 min at 4 °C. The pH of the clarified supernatant was immediately measured in triplicate, and the average value was recorded.

### DNA extraction

Total genomic DNA was extracted from swab and muscle tissue samples using the E.Z.N.A.^®^ Soil DNA Kit (Omega Bio-tek, USA) and from intestinal content samples using the E.Z.N.A.^®^ Stool DNA Kit (Omega Bio-tek, USA), following the manufacturer’s protocols. DNA concentration and purity were assessed using a NanoDrop spectrophotometer Nano-800+ (Shanghai Jinpeng Analysis Instrument, China), and integrity was verified by 1% agarose gel electrophoresis.

### 16S rRNA gene amplicon sequencing

The V3-V4 hypervariable region of the bacterial 16S rRNA gene was amplified using barcoded primers 341F (5’-CCTAYGGGRBGCASCAG-3’) and 806R (5’-GGACTACNNGGGTATCTAAT-3’). PCR amplification was performed in a 30 µL reaction mixture containing 15 µL of Phusion High-Fidelity PCR Master Mix (New England Biolabs, USA), 0.2 µM of each primer, and approximately 10 ng of template DNA. Thermal cycling conditions consisted of an initial denaturation at 98 °C for 1 min, followed by 30 cycles of denaturation at 98 °C for 10 s, annealing at 50 °C for 30 s, and extension at 72 °C for 30 s, with a final extension at 72 °C for 5 min. PCR products were purified using magnetic bead-based purification, quantified by Qubit fluorometry (Thermo Fisher Scientific, USA), and pooled in equimolar ratios. Sequencing libraries were constructed with index tags, quality-checked using a Bioanalyzer (Agilent Technologies, USA), and sequenced on the Illumina NovaSeq 6000 with paired-end 250 bp reads at Novogene (Beijing, China), generating approximately 10,000 paired-end sequences per sample.

Five representative time points (0, 24, 40, 56, and 64 h) were selected for 16S rRNA gene amplicon sequencing to capture critical stages of microbial community succession during spoilage progression of grass carp. A total of 50 samples were subjected to sequencing analysis, comprising muscle tissue samples (*n* = 26) and intestinal content samples (*n* = 24), with two biological replicates per group. Surface samples were analyzed at all five time points. Intestinal samples at 0 h were excluded from analysis due to unsuccessful PCR amplification, attributed to insufficient DNA quantity, with most extracted nucleic acids prioritized for metagenomic sequencing. Therefore, gut microbiota analysis commenced at 24 h. The detailed information of the samples subjected to 16S rRNA gene amplicon sequencing is provided in Table S1.

### Bioinformatics analysis of 16S rRNA gene amplicon sequencing data

Raw paired-end reads were demultiplexed based on unique barcodes and quality-filtered using fastp (v0.24.3) [14] to remove low-quality sequences and adapter contamination. The resulting clean sequences were denoised into amplicon sequence variants (ASVs) using the DADA2 plugin in QIIME2 (v2025.7) [15]. Taxonomic assignment was performed against the SILVA 138.1 SSU Ref NR99 database using the feature-classifier plugin in QIIME2 with a 97% similarity threshold. To identify potential bacterial pathogens, representative sequences of ASVs were aligned against the bacterial pathogen 16S rRNA gene collection in the MBPD [16] using BLASTn (v2.17.0+). Pathogenic bacteria were identified based on the threshold criteria of ≥ 90% sequence similarity and ≥ 90% query coverage. Alpha diversity indices, including Shannon and Pielou’s evenness, were calculated using QIIME2. Beta diversity was evaluated based on weighted UniFrac distance matrices and visualized through principal coordinate analysis (PCoA). Permutational multivariate analysis of variance (PERMANOVA) was performed to test the significance of packaging types, sampling site, and storage time effects on bacterial community structure. Similarity percentage analysis (SIMPER) was conducted to identify bacterial taxa contributing to community dissimilarity between groups, and the top ten taxa with the highest cumulative contributions are reported. Linear discriminant analysis effect size (LEfSe) was performed to identify differentially abundant bacterial taxa among treatment groups, with an LDA score > 2.0 and *p* < 0.05. Spearman’s rank correlation analysis was conducted to assess relationships between dominant bacterial genera and physicochemical parameters. Distance-based redundancy analysis (dbRDA) was performed to evaluate the contribution of physicochemical factors to bacterial community variation.

### Metagenomic sequencing and analysis

Nine representative surface swab and intestinal content samples were selected for shotgun metagenomic sequencing, as detailed in Table S2. Metagenomic sequencing libraries were constructed using the NEBNext^®^ Ultra™ DNA Library Prep Kit for Illumina (New England Biolabs, USA) following the manufacturer’s protocol. Library quality was assessed using a Bioanalyzer (Agilent Technologies, USA), and quantified libraries were pooled and sequenced on the Illumina NovaSeq 6000 platform with paired-end 150 bp reads at Novogene (Beijing, China), generating approximately 10 Gb of raw data per sample.

Raw metagenomic reads were quality-filtered using fastp (v0.24.3). High-quality clean reads were analyzed for ARGs using the ARGs-OAP (v3.2.4) [17] against the SARG database. ARG abundance was normalized to per copy of the 16S-rRNA gene and classified by antibiotic resistance type.

### Statistical analysis

All statistical analyses and graphical representations were performed using R software (v4.4.1). To assess differences among multiple independent groups, the Kruskal-Wallis one-way analysis of variance was applied as a nonparametric alternative. In cases where significant group effects were observed, post hoc pairwise comparisons were conducted using the Wilcoxon signed-rank test, with *p*-values adjusted for multiple comparisons. Statistical significance was defined as *p* < 0.05 throughout the study.

## Results and discussion

### Spoilage progress of grass carp under simulated household packaging conditions

This study investigated the spoilage dynamics of whole grass carp under simulated household storage conditions using three common packaging scenarios: Group A (sealed PE bags), Group B (unsealed PE bags with partial air exchange), and Group C (unpacked fish with full environmental exposure). Storage was conducted at room temperature with mean values of 13.0 ± 3.4 °C and 43.9 ± 10.8% relative humidity over the 72-hour experimental period (Fig. S1a). These conditions simulate typical domestic storage environments in regions with limited or intermittent refrigeration access, where consumers may store fresh fish for short periods before consumption or further processing.

**Fig. 1:**
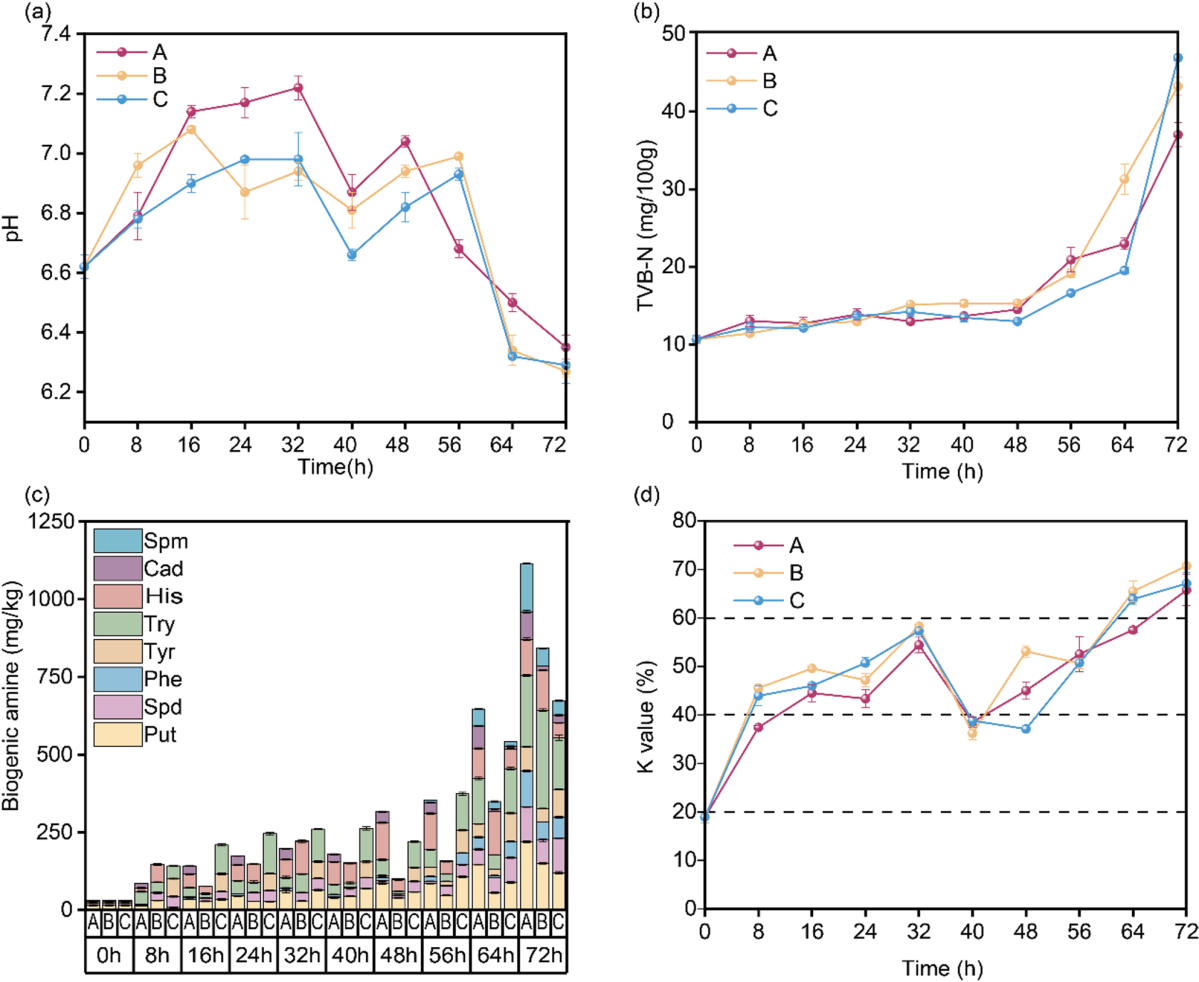
Temporal dynamics of physicochemical parameters in grass carp muscle under different conventional packaging conditions. **a** pH variation. **b** TVB-N accumulation; **c** Biogenic amine accumulation. **d** K value variation. Group A: fish in sealed PE bags; Group B: fish in unsealed PE bags with partial air exchange; Group C: unpacked fish with full environmental exposure. Cad, Cadaverine; Phe, Phenylethylamine; His, Histamine; Try, Tryptamine; Tyr, Tyramine; Spm, Spermine; Spd, Spermidine; Put, Putrescine

An inverted U-shaped pattern was observed in the pH profiles of grass carp muscle tissue under all packaging treatments during the 72-hour storage period (Fig. 1a). Initially, pH increased from approximately 6.7 to peak values of 7.0 ~ 7.2 at 32 h, followed by a pronounced decline to about 6.29 ~ 6.35 by the endpoint. This pattern aligns with the classical understanding of fish spoilage biochemistry, wherein early-stage alkalinization results from microbial decarboxylase and deaminase activities producing volatile basic nitrogen compounds, including ammonia, trimethylamine (TMA), and biogenic amines derived from free amino acid catabolism. The subsequent acidification phase reflects the accumulation of organic acids (e.g., lactic acid, acetic acid, and formic acid) generated through fermentative metabolism and carbohydrate breakdown by spoilage-associated bacteria [18]. Notably, Group A exhibited the highest peak pH and most delayed acidification, suggesting that sealed PE bags with restricted oxygen availability favor amino acid decarboxylation over aerobic conditions. Conversely, Group C (unpacked) showed earlier pH decline, potentially due to enhanced aerobic respiration facilitating rapid organic acid production by bacteria such as *Pseudomonas* and *Shewanella* species, which are known to thrive under high-oxygen conditions [19].

TVB-N is a widely accepted chemical index for assessing seafood spoilage, integrating ammonia, TMA, dimethylamine, and other volatile nitrogenous bases derived from protein degradation. As shown in Fig. 1b, TVB-N concentrations increased gradually during the initial 48 h (13.0 ~ 15.3 mg/100 g), followed by exponential accumulation reaching 37.0 ~ 47.0 mg/100 g at 72 h, substantially exceeding the Chinese national safety limit of 20 mg/100 g for fresh fish (GB 2733 − 2005).

As shown in Fig. S1b, the total sulfhydryl content significantly decreased (*p* < 0.05) from 8.75 ± 0.33 µmol/g to 6.15 ± 0.67 µmol/g over the 72-hour storage period. This reduction indicates progressive protein oxidation, a critical degradation pathway that impairs texture, water-holding capacity, and nutritional quality in fish muscle. Total sulfhydryl content, primarily derived from cysteine and methionine residues in myofibrillar proteins, is a sensitive indicator of oxidative damage, involving mechanisms such as thiol-disulfide exchange and sulfinic/sulfonic acid formation [20]. Our results demonstrate that grass carp underwent significant protein oxidation under ambient storage, particularly after 48 h.

Biogenic amines are low-molecular-weight nitrogen-containing compounds formed primarily through bacterial decarboxylation of free amino acids, and their accumulation poses both organoleptic defects (off-odors, bitter taste) and potential health risks (diarrhea, food poisoning, vomiting, sweating, or tachycardia) [21]. The quantitative biogenic amine profiles (Fig. 1c) demonstrated progressive accumulation of eight major biogenic amines over the 72 h, with putrescine (Put), tryptamine (Try), and spermidine (Spd) emerging as dominant species at endpoint concentrations exceeding 100, 150, and 40 mg/kg, respectively. The biogenic amine index (BAI = His + Put + Cad + Tyr) serves as a comprehensive freshness indicator, with values < 50 mg/kg defining acceptable sensory quality [22]. The BAI increased from an initial 26.9 mg/kg to significantly elevated levels after 72 h of storage (Group A: 501.5 mg/kg, Group B: 334.8 mg/kg, Group C: 281.0 mg/kg). Notably, the BAI in all three groups exceeded the 50 mg/kg threshold as early as 16 h, indicating a marked loss of freshness in the fish. Group A accumulated the highest total biogenic amine (1114.9 mg/kg) and biogenic amine index (501.5 mg/kg), reflecting oxygen-restricted conditions favoring facultative anaerobes with diverse decarboxylase activities [23].

ATP content declined progressively from 127.98 ~ 193.19 µmol/g at 0 ~ 8 h to 7.74 ~ 11.35 µmol/g at 72 h, reflecting rapid post-mortem energy depletion, while ADP remained relatively stable at 8.51 ~ 16.45 µmol/g throughout storage (Fig. S2a, b). IMP exhibited biphasic behavior, increasing from 491.63 µmol/g initially to peak at 677.12 ~ 764.49 µmol/g around 40 h before declining to 440.41 ~ 593.10 µmol/g by 72 h (Fig. S2d), reflecting initial accumulation via AMP deamination followed by degradation to HxR and Hx through endogenous and microbial enzymes [24]. Hx and HxR levels showed a sharp increase between 64 and 72 h compared to the baseline (0 h) (Fig. S2e, f). To quantify the freshness of fish samples based on post-mortem energy metabolism, the K value is employed as a biochemical index. This value is defined as the molar percentage of HxR and Hx in relation to the total pool of ATP and its degradation intermediates. During storage, the K value progression reflected distinct freshness transitions (Fig. 1d). From 0 ~ 8 h, fish remained fresh. Between 16 ~ 56 h, K values rose from 36.2% to 57.3%, marking complete freshness loss and entry into early spoilage. By 64 ~ 72 h, K values reached 65.7 ~ 70.7%, indicating advanced spoilage with severe nucleotide degradation. This temporal pattern aligns closely with TVB-N escalation and biogenic amine accumulation, confirming synchronized biochemical deterioration.

This finding has critical implications for rural/tropical regions lacking cold chain infrastructure. Our findings demonstrate that grass carp stored under ambient household conditions maintained acceptable freshness (K < 40%, BAI < 50 mg/kg) for less than16 hours, transitioning to marginal quality (K 40 ~ 60%) by 48 h and complete spoilage (K > 60%, TVB-*N* > 20 mg/100 g) by 64 h. This rapid deterioration necessitates: (1) immediate consumption within 16 h post-purchase; (2) adoption of traditional preservation methods (salting, smoking, fermentation) for extended storage; or (3) implementation of affordable cold chain alternatives (evaporative cooling, ice supplementation). While sealed PE bags provided marginal benefits in delaying TVB-N accumulation and protein oxidation, they cannot substitute for refrigeration in ensuring food safety and quality retention.

### Microbial community succession during grass carp spoilage

We conducted 16S rRNA amplicon sequencing throughout the 72-hour storage period to characterize microbial community dynamics in grass carp muscle and gut samples across three packaging treatments. The phylogenetic analysis (Fig. S3) revealed high taxonomic diversity among microbes in fish muscle and gut, encompassing 17 bacterial phyla with Pseudomonadota, Bacteroidota, Bacillota, and Actinomycetota as dominant lineages. Fresh muscle samples (CS00) exhibited significantly lower diversity and evenness compared to all storage time points, reflecting initial oligotrophic conditions (Fig. S4). Within 24 h, Shannon diversity and Pielou’s evenness dramatically increased, indicating rapid colonization by diverse spoilage-associated taxa following breakdown of host antimicrobial defenses.

**Fig. 2:**
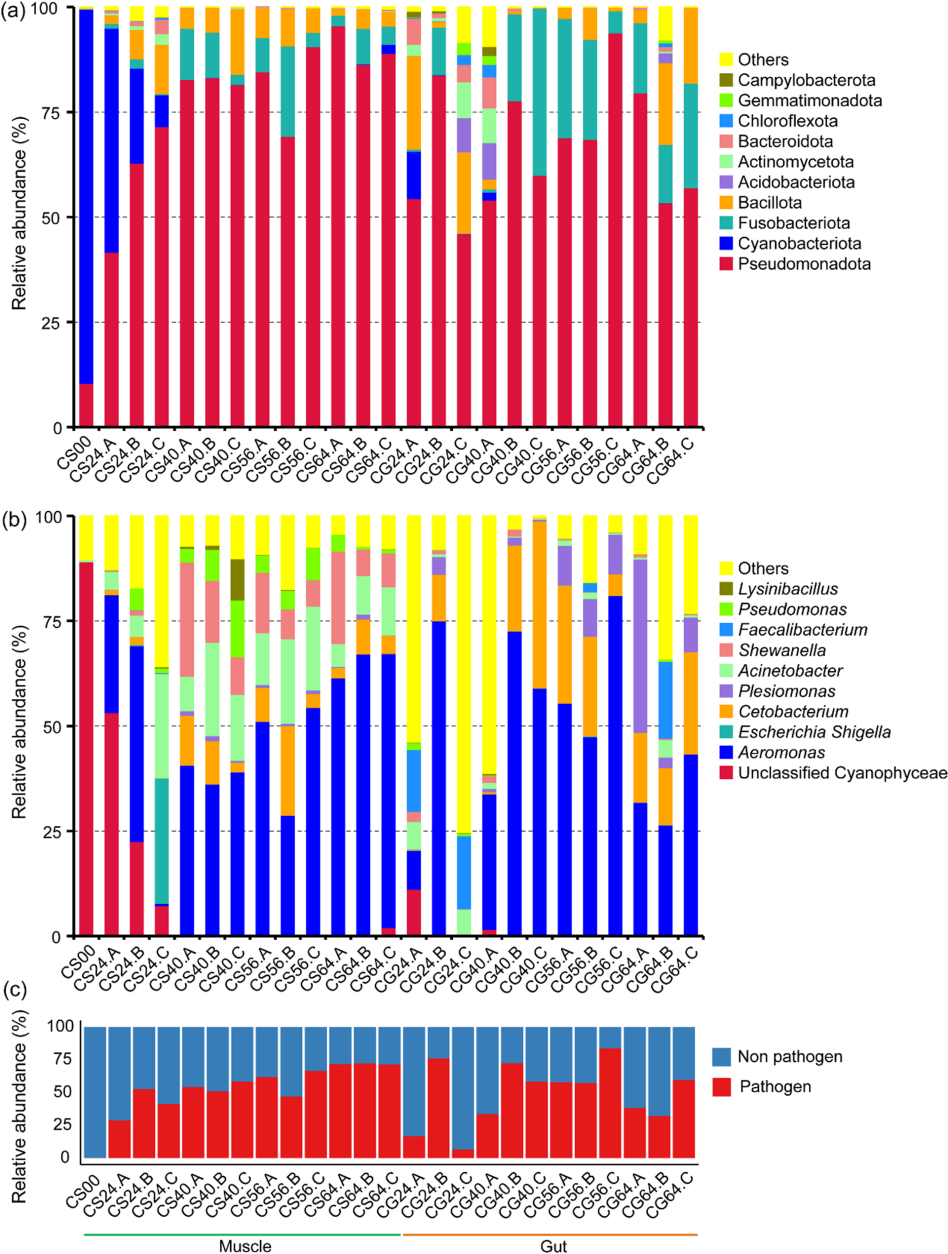
Dynamics of dominant bacterial taxa in grass carp muscle tissue and gut samples during storage under different conventional packaging. **a** Temporal changes in bacterial composition at the phylum level (*n* = 2). **b** Temporal changes at the genus level (*n* = 2). **c** Dynamics of potential pathogens

Muscle tissue microbial communities underwent stage-specific shifts correlated with packaging treatments and storage time (Fig. 2). Fresh muscle samples (CS00) were dominated by Cyanobacteriota (89.1 ± 4.3%, primarily unclassified Cyanophyceae) with minimal Pseudomonadota (10.4 ± 4.0%). This low-diversity profile represents initial oligotrophic conditions with limited bacterial colonization on fish muscle. The significant proportion of unclassified Cyanophyceae suggests the presence of novel cyanobacterial lineages in freshwater fish ecosystems. This pattern can be attributed to either a prevalence of truly novel taxa awaiting discovery or to current database limitations against microbes in fish-related environments. Consequently, genome-level characterization is required to obtain higher taxonomic resolution and distinguish between these scenarios. Upon storage initiation, Cyanobacteriota rapidly declined to < 1%, while Pseudomonadota expanded dramatically to 41.7 ~ 95.6% throughout 24 ~ 72 hours, demonstrating superior competitive fitness under ambient storage conditions. Pseudomonadota encompasses major SSO genera (*Acinetobacter*, *Pseudomonas*, *Shewanella*, and *Aeromonas*) capable of utilizing diverse nitrogen sources like amino acids, nucleotides, and trimethylamine N-oxide (TMAO) and producing volatile spoilage metabolites (ammonia, TMA, sulfides) in fish [7, 25, 26]. At the genus level, *Aeromonas*, *Acinetobacter*, *Shewanella*, and *Pseudomonas* were abundant and increased progressively over time in fish muscle during storage (Fig. 2b). The expansion of Pseudomonadota was primarily driven by *Aeromonas*, which emerged as the dominant spoilage-related organism throughout storage. Initially present at only 0.001% in fish muscle at 0 h, the relative abundance of *Aeromonas* increased progressively over time, reaching 61.4%~67.2% after 64 hours of storage. This dramatic increase reflects the superior competitive fitness of *Aeromonas* at ambient temperature. *Aeromonas* has been reported to possess versatile metabolic capabilities, including amino acid decarboxylase activities that produce biogenic amines, extracellular protease secretion for protein degradation, and 5’-nucleotidase involved in ATP catabolism [27, 28]. *Acinetobacter* served as a transient early colonizer within 24 h (4.3 ~ 24.8%), achieving the highest abundance during the early spoilage stage, followed by a decline during advanced spoilage. This biphasic pattern suggests opportunistic growth during the initial aerobic phase with abundant oxygen and nutrient availability, followed by competitive exclusion as *Aeromonas* established metabolic dominance through superior proteolytic and decarboxylase activities [11]. Pathogen abundance in muscle samples was negligible initially (0.06 ± 0.04%) but increased markedly to 28.8 ~ 52.7% within 24 h. A further progressive increase to 54.1 ~ 66.6% was observed at 40 ~ 56 h, followed by stabilization at 71.4 ~ 72.2% in the advanced spoilage phase (64 h) (Fig. 2c). This progressive pathogen accumulation, primarily comprising *Escherichia Shigella*, *Aeromonas*, and *Acinetobacter*, indicated substantial escalation of food safety risks as spoilage progressed.

Intestinal samples exhibited distinct compositional patterns compared to muscle tissues. Pseudomonadota dominated (45.9 ~ 93.9%), primarily driven by *Aeromonas* with an average abundance of 44.5%. Fusobacteriota maintained significantly higher abundance (*p* < 0.05) than muscle tissues, with its member *Cetobacterium* averaging 15.3% as a dominant gut commensal. *Faecalibacterium* belonging to Bacillota, an obligate anaerobic commensal, persisted at 14.7 ~ 18.2% in specific gut samples (CG24.A, CG24.C, and CG64.B) but was completely absent in muscle tissues. At 24 h, pathogen abundance varied considerably across samples, ranging from 6.6% to 76.0%. Sample CG24.B exhibited the highest early pathogen load (76.0 ± 9.6%), which was consistent with a marked dominance of opportunistic pathogen *Aeromonas* (74.9 ± 8.4%). In contrast, samples CG24.A and CG24.C maintained significantly lower pathogen levels (16.7 ± 3.6% and 6.6 ± 5.8%, respectively). These results indicate that as spoilage progressed beyond 40 h, pathogenic bacteria increasingly dominated the intestinal communities, underscoring the persistent food safety risks during advanced spoilage.

### Community structure differentiation revealed by beta diversity analysis

**Fig. 3:**
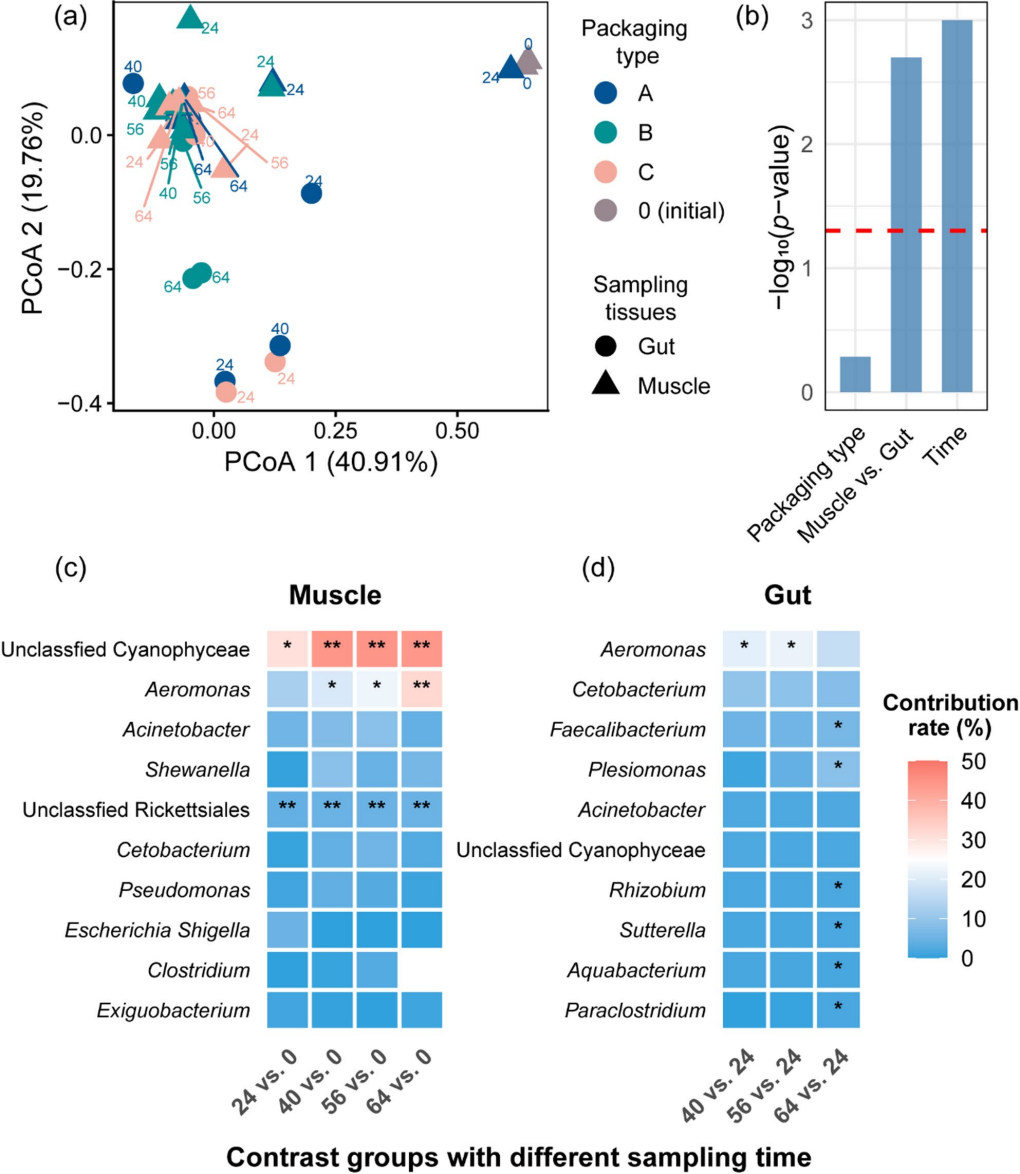
Beta diversity analysis of bacterial communities in grass carp under different packaging conditions. **a** PCoA based on weighted UniFrac dissimilarity showing separation among groups. **b** PERMANOVA results showing the significance of packaging type, sampling site, and storage time effects. The red dashed line indicates *p* = 0.05. **c** Bacterial taxa (top 10) at the genus level contributing to community dissimilarity across storage stages in muscle tissue samples identified by SIMPER analysis. **d** Bacterial taxa at the genus level contributing to community dissimilarity across storage stages in gut samples identified by SIMPER analysis. *, *p* < 0.05; **, *p* < 0.01; ***, *p* < 0.001

Principal coordinates analysis (PCoA) based on weighted UniFrac distances was conducted to analyze clustering patterns of bacterial communities across storage conditions, sampling time, and sampling tissues (Fig. 3a). Fresh muscle samples (CS00) clustered separately from all storage time points, reflecting fundamental compositional differences between initial oligotrophic conditions and post-storage microbial community. Along PC1 (40.91% variance), samples exhibited clear temporal progression from fresh state toward spoilage-associated communities, with 24-hour samples positioned intermediately between fresh and later storage stages. Gut samples and muscle surface samples displayed distinct clustering patterns, indicating tissue-specific microbial community differentiation. Notably, samples converged toward similar compositional states during advanced spoilage (56 ~ 64 h), particularly along PC2 (19.76% variance), suggesting convergent succession toward *Aeromonas*-dominated communities regardless of packaging treatment. PERMANOVA quantitatively confirmed the relative importance of different factors shaping community structure (Fig. 3b). Storage time was the most significant driver of microbial community variation (*p* < 0.001), explaining the progressive temporal shifts observed in PCoA ordination and demonstrating that spoilage-associated succession follows time-dependent deterministic trajectories. Sampling tissues (muscle vs. gut) showed significant influence (*p* < 0.05), confirming substantial tissue-specific microbial community differentiation, where gut communities retained higher compositional heterogeneity due to native commensal persistence (*Cetobacterium* and *Faecalibacterium*), while muscle tissues exhibited more uniform *Aeromonas*-dominated succession. In contrast, packaging type exhibited no significant effect on overall community structure (*p* > 0.05), indicating that packaging treatments (A, B, C) exerted minimal influence compared to intrinsic temporal and spatial factors.

SIMPER analysis identified key bacterial genera responsible for compositional shifts across storage stages in both muscle and gut tissues (Fig. 3c, d). In muscle tissue samples (Fig. 3c), unclassified Cyanophyceae, *Aeromonas*, and unclassified Rickettsiales significantly contributed to compositional shifts, representing potential biomarkers for spoilage progression. Unclassified Cyanophyceae exhibited the highest contribution rates (30.7 ~ 44.6%) during early storage transitions (*p* < 0.05), reflecting the dramatic decline of Cyanobacteriota-dominated fresh fish microbiota. *Aeromonas* showed progressive increase in contribution from fresh to advanced spoilage stages, with contribution rates escalating from 12.5% at 24 h (*p* > 0.05) to 32.3% at 64 h (*p* < 0.001), demonstrating its emergence as the dominant spoilage-specific organism. Unclassified Rickettsiales maintained consistently high contribution rates (4.2 ~ 4.8%) with significant differences across most temporal comparisons (*p* < 0.01), indicating persistent compositional influence throughout storage despite lower absolute abundance. LEfSe analysis revealed treatment-specific biomarker taxa despite overall *Aeromonas-*dominated succession (Fig. S5). Group A showed *Shewanella* enrichment, while group B exhibited diverse biomarkers spanning Pseudomonadales, *Acinetobacter*, and *Pseudomonas*, indicating that packaging conditions exerted subtle selective pressures on subordinate taxa and created treatment-specific compositional signatures. In gut samples (Fig. 3d), compositional dynamics exhibited greater complexity. *Aeromonas* remained the primary driver of temporal shifts with the highest contribution rates. Native gut commensals, including *Cetobacterium* and *Faecalibacterium*, showed substantial contributions to community dissimilarity. *Faecalibacterium*, *Plesiomonas*, *Rhizobium*, *Sutterella*, *Aquabacterium*, and *Paraclostridium* significantly contributed to the difference of 64 h vs. 24 h (*p* < 0.05). The contrasting succession patterns between muscle and gut tissues reflect fundamental ecological differences: muscle tissues undergo deterministic *Aeromonas*-dominated succession driven by proteolytic capacity and metabolic versatility [27, 28], while gut communities exhibit compositional resistance mediated by native commensals (*Cetobacterium*, *Faecalibacterium*) through competitive exclusion and niche occupation. These patterns demonstrate that microbial succession during grass carp spoilage is primarily governed by storage time-dependent processes, with tissue-specific constraints and the tested packaging conditions exerting modulating effects on community assembly trajectories.

### Environmental drivers of microbial community succession during grass carp spoilage

**Fig. 4:**
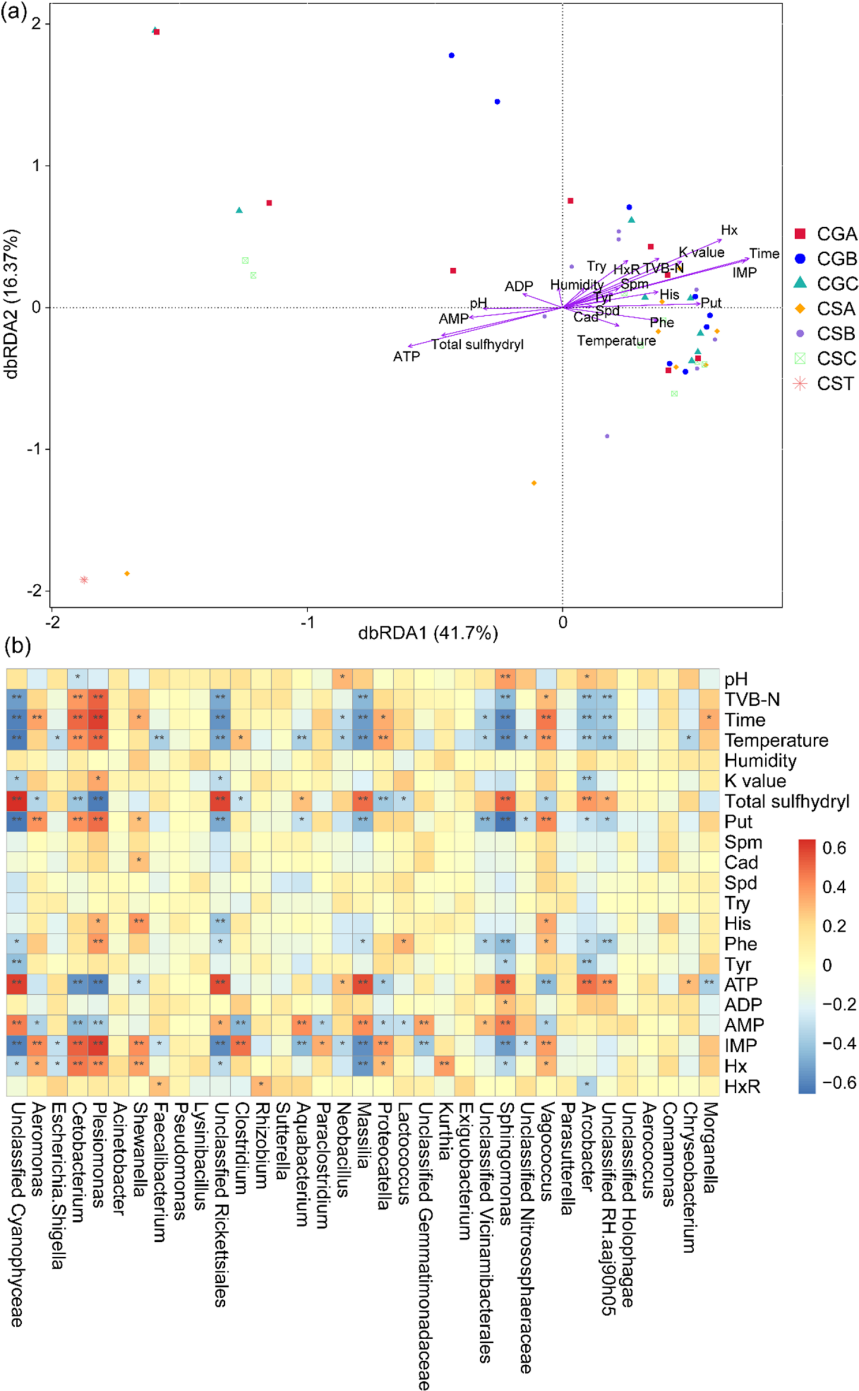
Relationships between bacterial communities and physicochemical parameters in grass carp under different packaging conditions. **a** Distance-based redundancy analysis (db-RDA) ordination showing correlations between bacterial community composition and environmental variables across packaging treatments. **b** Spearman correlation heatmap depicting significant associations between dominant genera and physicochemical parameters. *, *p* < 0.05; **, *p* < 0.01; ***, *p* < 0.001. CGA, group A of gut samples; CGB, group B of gut samples; CGC, group C of gut samples; CSA, group A of muscle samples; CSB, group B of muscle samples; CSC, group C of muscle samples; CST, initial muscle sample at 0 h

Distance-based redundancy analysis (db-RDA) revealed that physicochemical parameters collectively explained substantial variation in bacterial community composition across packaging treatments and storage times (Fig. 4a). The first two constrained axes captured 58.1% of the total variation in community-environment relationships, with samples clearly segregated along temporal and treatment-specific trajectories. Fresh muscle samples (CST) clustered distinctly in the lower-left quadrant, separated from all storage samples, reflecting their unique oligotrophic microbial profile dominated by Cyanobacteriota. This dramatic spatial segregation demonstrates the fundamental transition from fresh state to spoilage-associated communities driven by the rapid breakdown of host antimicrobial defenses and nutrient release. Temporal progression along RDA1 represented the primary gradient of spoilage succession (Fig. 4a). Early-stage samples (24 h) occupied intermediate positions, while mid-to-late stage samples (40 ~ 64 h) progressively shifted toward positive RDA1 values, corresponding to increasing HxR, Hx, TVB-N, and biogenic amine accumulation. This directional trajectory demonstrates that progressive protein degradation and TAP reduction, primarily mediated by *Aeromonas*, drove deterministic shifts in community composition toward SSO dominance [28]. RDA2 captured treatment-specific and tissue-specific variation orthogonal to temporal succession. Gut samples (CGA, CGB, CGC) displayed greater dispersion along RDA2 compared to muscle samples within corresponding time points, reflecting heterogeneous bacterial dynamics modulated by native commensal bacteria such as *Cetobacterium* and *Faecalibacterium*. Packaging treatments induced subtle but discernible compositional shifts along treatment-specific trajectories. At the early stage (24 h), CSC samples (environmental exposure) consistently occupied more positive RDA1 positions compared to CSA/CSB (packaging) at equivalent time points. The convergent trajectories of all treatments toward the upper-right quadrant at 56 ~ 64 h demonstrate that despite packaging-modulated early succession, late-stage communities universally converged toward *Aeromonas* dominance driven by its superior competitive fitness under ambient storage conditions.

Spearman correlation analysis complemented db-RDA by quantifying genus-level responses to individual physicochemical parameters (Fig. 4b). *Aeromonas* and *Shewanella*, the predominant SSOs, exhibited strong positive correlations with storage time, Put, IMP, and Hx across treatments (*p* < 0.05), demonstrating their central roles in ATP catabolism and biogenic amine biosynthesis. TVB-N accumulation was significantly associated with *Cetobacterium*, *Plesiomonas*, and *Vagococcus* (*p* < 0.05), indicating these genera contribute substantially to protein degradation and ammonia/trimethylamine production despite their lower relative abundances.

### ARG dynamics during grass carp spoilage

**Fig. 5:**
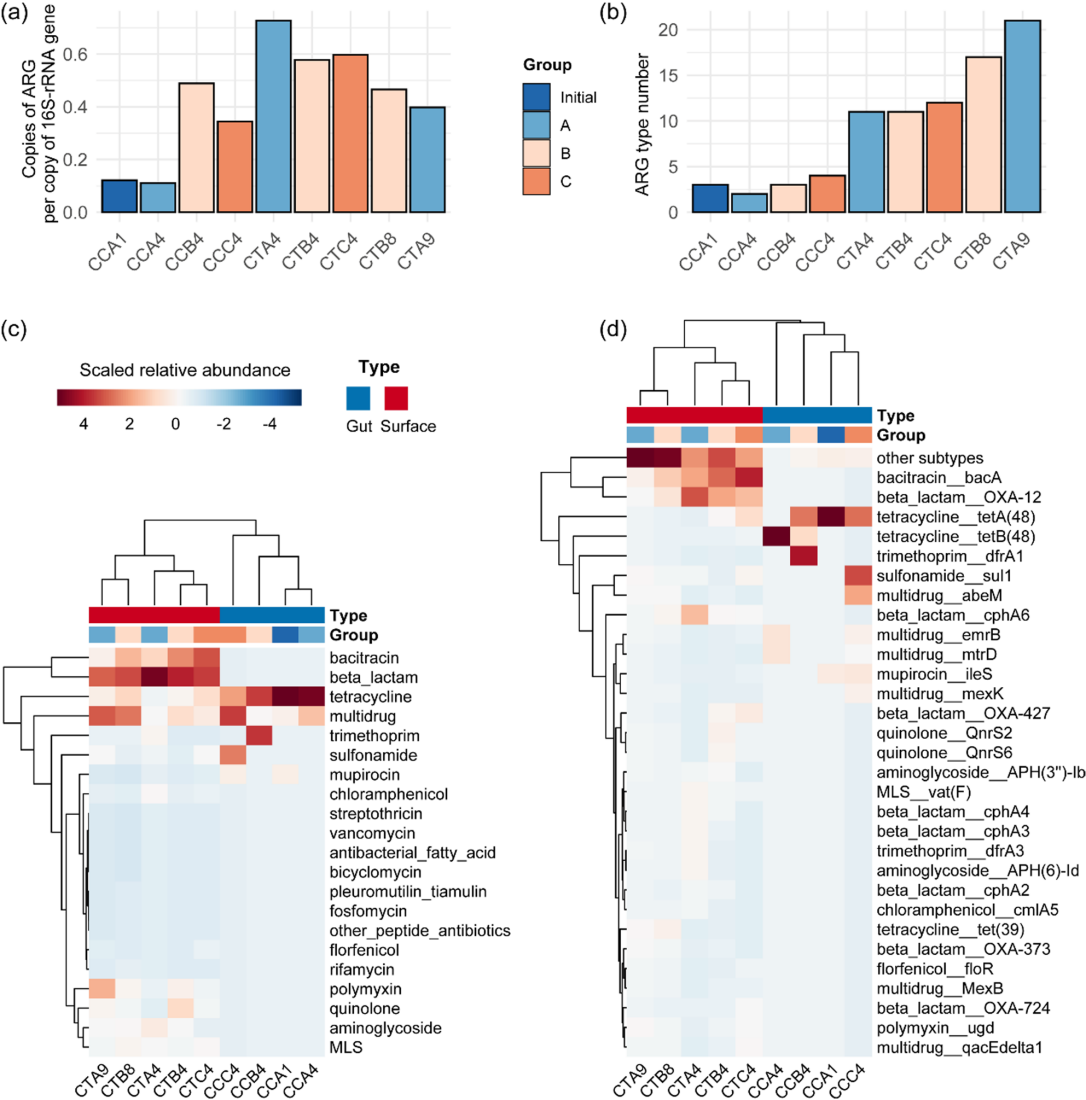
Abundance and composition of ARGs in surface and gut samples of grass carp during storage under different packaging conditions. **a** Relative abundance of ARGs normalized to 16S rRNA gene copies. **b** Number of ARG types in each sample. **c** Heatmap and hierarchical clustering of ARG categories across sample types (gut and skin) and groups (A, B, C). **d** Heatmap and hierarchical clustering of individual ARG subtypes. Microbial samples from the gut include CCA1, CCA4, CCB4, and CCC4. Microbial samples from fish surface include CTA4, CTB4, CTC4, CTB8, and CTA9

The skin and gut microbial samples from grass carp were subjected to metagenomic sequencing to assess the ARG risk during fish spoilage. Total ARG abundance normalized to 16S rRNA gene copies rose from 0.121 in fresh gut samples (CCA1, 0 h) to 0.11 ~ 0.49 copies/16S rRNA gene in gut samples at 24 h (CCA4, CCB4, CCC4), with Group B showing the highest gut ARG load (Fig. 5a). Surface samples exhibited dramatically higher ARG abundances, reaching 0.58 ~ 0.73 copies/16S rRNA gene at 24 h across all groups. This tissue-specific pattern reflects the skin microbes’ greater exposure to environmental stressors, which might induce the reproduction of resistant populations [29].

The baseline intestinal resistome of fresh gut samples (CCA1) was characterized by a limited diversity of only three antibiotic resistance gene (ARG) types: tetracycline (0.10 copies/16S rRNA), multidrug (0.01), and mupirocin (0.01) (Fig. 5b-d). The specific ARGs included *tet*A(48), *mex*N, *Mux*B, and *ile*S. At 24 h, gut samples diverged dramatically by groups: CCB4 (Group B) showed dominant tetracycline (0.228) and trimethoprim (0.237) resistance, CCC4 (Group C) exhibited elevated multidrug (0.130) and sulfonamide (0.101) resistance, while CCA4 (Group A) maintained a profile similar to fresh samples with tetracycline (0.082) and multidrug (0.029) genes predominating. Notably, enrichment of the ARGs *tet*A(48), *tet*B(48), *sul1*, *dfr*A1, and *abe*M was observed in gut samples at 24 h.

Surface samples at 24 h displayed comprehensive ARG repertoires spanning 11 ~ 21 resistance types. Beta-lactam resistance genes dominated all surface samples, followed by bacitracin, multidrug, tetracycline, polymyxin, aminoglycoside, macrolide-lincosamide-streptogramin (MLS), sulfonamide, and chloramphenicol resistance. Advanced spoilage samples (CTB8 and CTA9) maintained diverse ARG profiles but with altered resistance type distributions compared to 24 h. Beta-lactam resistance remained prominent, while tetracycline, multidrug, and polymyxin resistance increased. Notably, CTA9 (64 h) showed the highest ARG type richness (21 types), encompassing nearly all detected resistance types. The emergence of novel resistance types, including fosfomycin, rifamycin, and pleuromutilin resistance in advanced spoilage samples, indicates progressive resistome diversification during extended storage [30]. At the subtype level, Bacitracin resistance *bac*A (0.06 ~ 0.17) was the most abundant ARG across all groups in surface samples. Notably, extensive ARG subtype diversity dominated by beta-lactamase variants was observed. OXA-type beta-lactamases OXA-12, OXA-427, OXA-724, and OXA-373 were the most abundant beta-lactam resistance genes across all groups. The detection of metallo-β-lactamases *cph*A3, *cph*A4, and *cph*A6 raises particular concern, as these enzymes confer resistance to carbapenems, last-resort antibiotics for treating multidrug-resistant Gram-negative infections [31]. The predominance of *Aeromonas* as the spoilage-associated genus in this study aligns with its recognized role as a reservoir of diverse ARGs in aquatic and food environments [32–34]. Some *Aeromonas* isolates belong to multidrug-resistant bacteria circulating in aquatic environments and the food production chain, which can potentially disseminate antimicrobial resistance to humans via the foodborne route [34]. Members of the genus *Aeromonas* are intrinsically resistant to β-lactams through the production of β-lactamases, including CphA and OXA-type enzymes [33]. Furthermore, *Aeromonas* isolates from aquaculture products and clinical settings frequently exhibit resistance to tetracyclines, sulfonamides, and fluoroquinolones [33]. The co-occurrence of clinically significant carbapenemase genes in a dominant spoilage organism underscores the potential role of fish microbiota as a reservoir of resistance determinants that could pose risks if transferred to human pathogens or acquired by mobile genetic elements.

The rapid ARG enrichment during early spoilage (24 h) represents a critical food safety concern, as fish may still be marketed at this stage despite harboring about 2.6-fold elevated ARG loads compared to fresh samples. Surface samples accumulated 11 ~ 12 ARG types within 24 h, expanding to 17 ~ 21 types by 56 ~ 64 h, with clinically important resistance determinants (carbapenem resistance, quinolone resistance, aminoglycoside resistance) reaching substantial abundances. While overtly spoiled fish is typically discarded, cross-contamination during handling, processing, and retail display could facilitate ARG transfer to food contact surfaces, food handlers’ microbiota, or co-occurring foodborne pathogens [35, 36]. The tissue-specific resistance profiles indicate that surface sanitation is more critical than evisceration for ARG load reduction. Surface microbial community harbored about 2-fold higher ARG abundance and 3.8-fold greater ARG type richness than gut samples at 24 h, establishing the fish surface as the primary resistance reservoir during ambient storage.

## Conclusions

This study establishes a comprehensive framework linking biochemical deterioration, microbial succession, and antibiotic resistance proliferation during grass carp spoilage under ambient household storage conditions, providing insights for food safety management in regions with limited cold chain infrastructure. We found that grass carp quality deteriorated rapidly under ambient storage, with acceptable freshness maintained for less than 16 h before the biogenic amine index exceeded safety thresholds, progressing to complete spoilage by 64 h. This narrow window of edibility challenges conventional household storage practices and necessitates immediate consumption or preservation interventions. Spoilage progression follows deterministic microbial succession trajectories dominated by *Aeromonas*, which emerges as the keystone specific spoilage organism. Critically, fish surfaces serve as primary reservoirs for ARG dissemination, accumulating clinically important carbapenem resistance determinants within 24 h at levels posing cross-contamination risks to food handlers, processing environments, and co-occurring foodborne pathogens. These findings have immediate practical implications. For resource-limited settings, traditional preservation methods (salting, smoking, fermentation) or affordable cold chain alternatives (ice supplementation, evaporative cooling) should be prioritized over extended ambient storage. For the seafood industry, surface sanitation protocols warrant greater emphasis than evisceration alone, as surface microbiota harbor 2.6-fold higher ARG loads and 3.8-fold greater resistance diversity than gut communities. For public health authorities, these results underscore the dual threat of spoiled seafood as both a foodborne illness vector and an antimicrobial resistance dissemination pathway, necessitating integrated surveillance frameworks.

## Supplementary Information

Supplementary Material 1.

## Data Availability

The sequencing data are available in the Genome Sequence Archive (GSA) of the National Genomics Data Center under accession number CRA037241 (https://ngdc.cncb.ac.cn/gsa/browse/CRA037241).
