# Supplementary material for: Microbial succession accompanies increased antibiotic resistance risk during grass carp (Ctenopharyngodon idella) spoilage under ambient household conditions
Source: BMC Microbiol. 2026 Mar 9;26:358. doi: 10.1186/s12866-026-04924-w (PMC13085627; doi:10.1186/s12866-026-04924-w)
Supplement: Supplementary file 1 — Supplementary Material 1. [file 12866_2026_4924_MOESM1_ESM.docx]

Supplementary information for

**Microbial Succession Accompanies Increased Antibiotic Resistance Risk during Grass Carp (*Ctenopharyngodon idella*) Spoilage under Ambient Household Conditions**

Zehan Shen^a,b,1^, Zuowu Zhang^a,1^, Jingrong Gao^b^, Jiao Chen^a^, Qiuxiang Xu^a^, Dongyi Li^a^, Li Zeng^a^, Dengmiao Cheng^a^, Kai Wang^a^, Jiayu Zhang^a,*^, Jonathan W.C. Wong^a,*^

^a^ Research Center for Eco-environmental Engineering, Dongguan University of Technology, Dongguan, 523808, China

^b^ School of Food and Pharmacy, Zhejiang Ocean University, Zhejiang, 316022, China

^*^ Corresponding author: Jiayu Zhang and Jonathan W.C. Wong

E-mail address: [zhangjiayu@dgut.edu.cn](mailto:zhangjiayu@dgut.edu.cn) (J.Y. Zhang), [jwcwong@dgut.edu.cn](mailto:jwcwong@dgut.edu.cn) (J.W.C. Wong)

^1^ Contributed equally to this work.

**This file includes:**

**Supplemental Tables：**

**Table S1.** Sample information for 16S rRNA gene amplicon sequencing.

**Table S2.** Sample information for metagenomic sequencing.

**Supplemental Figures：**

**Fig. S1.** Environmental parameters and protein oxidation in grass carp during storage under different packaging conditions.

**Fig. S2.** Dynamics of ATP and its degradation products in grass carp muscle during storage under different packaging conditions.

**Fig. S3.** Phylogenetic relationships of dominant bacterial genera with abundance profiles across three packaging treatments during storage.

**Fig. S4.** Alpha diversity (Shannon and Pielou’s evenness indices) of muscle tissue and gut bacterial communities in grass carp under different packaging conditions.

**Fig. S5.** LEfSe analysis of differential bacterial taxa in muscle tissue and gut samples of grass carp under different packaging conditions.

**Table S1. Sample information for 16S rRNA gene amplicon sequencing.**

| **Sample ID** | **Group** | **Time (h)** | **Site** | **Packaging treatment** | **Raw reads** |
| --- | --- | --- | --- | --- | --- |
| CS00.A.r1 | CS00.A | 0 | Muscle | - | 128201 |
| CS00.A.r2 | CS00.A | 0 | Muscle | - | 104816 |
| CG24.A.r1 | CG24.A | 24 | Gut | A | 103620 |
| CG24.A.r2 | CG24.A | 24 | Gut | A | 120204 |
| CG24.B.r1 | CG24.B | 24 | Gut | B | 103324 |
| CG24.B.r2 | CG24.B | 24 | Gut | B | 133063 |
| CG24.C.r1 | CG24.C | 24 | Gut | C | 105801 |
| CG24.C.r2 | CG24.C | 24 | Gut | C | 136552 |
| CS24.A.r1 | CS24.A | 24 | Muscle | A | 105291 |
| CS24.A.r2 | CS24.A | 24 | Muscle | A | 103176 |
| CS24.B.r1 | CS24.B | 24 | Muscle | B | 67167 |
| CS24.B.r2 | CS24.B | 24 | Muscle | B | 110999 |
| CS24.C.r1 | CS24.C | 24 | Muscle | C | 96144 |
| CS24.C.r2 | CS24.C | 24 | Muscle | C | 102711 |
| CG40.A.r1 | CG40.A | 40 | Gut | A | 129561 |
| CG40.A.r2 | CG40.A | 40 | Gut | A | 122408 |
| CG40.B.r1 | CG40.B | 40 | Gut | B | 102808 |
| CG40.B.r2 | CG40.B | 40 | Gut | B | 72604 |
| CG40.C.r1 | CG40.C | 40 | Gut | C | 83890 |
| CG40.C.r2 | CG40.C | 40 | Gut | C | 92566 |
| CS40.A.r1 | CS40.A | 40 | Muscle | A | 105199 |
| CS40.A.r2 | CS40.A | 40 | Muscle | A | 102644 |
| CS40.B.r1 | CS40.B | 40 | Muscle | B | 105949 |
| CS40.B.r2 | CS40.B | 40 | Muscle | B | 102079 |
| CS40.C.r1 | CS40.C | 40 | Muscle | C | 104590 |
| CS40.C.r2 | CS40.C | 40 | Muscle | C | 153610 |
| CG56.A.r1 | CG56.A | 56 | Gut | A | 72472 |
| CG56.A.r2 | CG56.A | 56 | Gut | A | 104114 |
| CG56.B.r1 | CG56.B | 56 | Gut | B | 105284 |
| CG56.B.r2 | CG56.B | 56 | Gut | B | 101894 |
| CG56.C.r1 | CG56.C | 56 | Gut | C | 107637 |
| CG56.C.r2 | CG56.C | 56 | Gut | C | 88584 |
| CS56.A.r1 | CS56.A | 56 | Muscle | A | 56599 |
| CS56.A.r2 | CS56.A | 56 | Muscle | A | 100026 |
| CS56.B.r1 | CS56.B | 56 | Muscle | B | 103671 |
| CS56.B.r2 | CS56.B | 56 | Muscle | B | 112564 |
| CS56.C.r1 | CS56.C | 56 | Muscle | C | 110931 |
| CS56.C.r2 | CS56.C | 56 | Muscle | C | 87991 |
| CG64.A.r1 | CG64.A | 64 | Gut | A | 103488 |
| CG64.A.r2 | CG64.A | 64 | Gut | A | 117580 |
| CG64.B.r1 | CG64.B | 64 | Gut | B | 103391 |
| CG64.B.r2 | CG64.B | 64 | Gut | B | 209027 |
| CG64.C.r1 | CG64.C | 64 | Gut | C | 110780 |
| CG64.C.r2 | CG64.C | 64 | Gut | C | 84433 |
| CS64.A.r1 | CS64.A | 64 | Muscle | A | 110289 |
| CS64.A.r2 | CS64.A | 64 | Muscle | A | 113448 |
| CS64.B.r1 | CS64.B | 64 | Muscle | B | 106253 |
| CS64.B.r2 | CS64.B | 64 | Muscle | B | 107273 |
| CS64.C.r1 | CS64.C | 64 | Muscle | C | 127805 |
| CS64.C.r2 | CS64.C | 64 | Muscle | C | 156779 |

Group A consisted of fish packed in PE bags sealed with twist ties; Group B comprised fish packed in PE bags without sealing to allow partial air exchange; and Group C included unpacked fish with full environmental exposure.

**Table S2. Sample information for metagenomic sequencing.**

| **Sample ID** | **Time (h)** | **Site** | **Packaging treatment** | **Sequencing depth (Gb)** | **Clean reads (M)** |
| --- | --- | --- | --- | --- | --- |
| CCA1 | 0 | Gut | - | 10.99 | 10.86 |
| CCA4 | 24 | Gut | A | 11.92 | 11.77 |
| CCB4 | 24 | Gut | B | 10.51 | 10.40 |
| CCC4 | 24 | Gut | C | 11.74 | 11.61 |
| CTA4 | 24 | Surface | A | 11.90 | 11.77 |
| CTB4 | 24 | Surface | B | 13.81 | 13.68 |
| CTC4 | 24 | Surface | C | 11.58 | 11.74 |
| CTB8 | 56 | Surface | B | 12.50 | 12.33 |
| CTA9 | 64 | Surface | A | 10.35 | 10.29 |


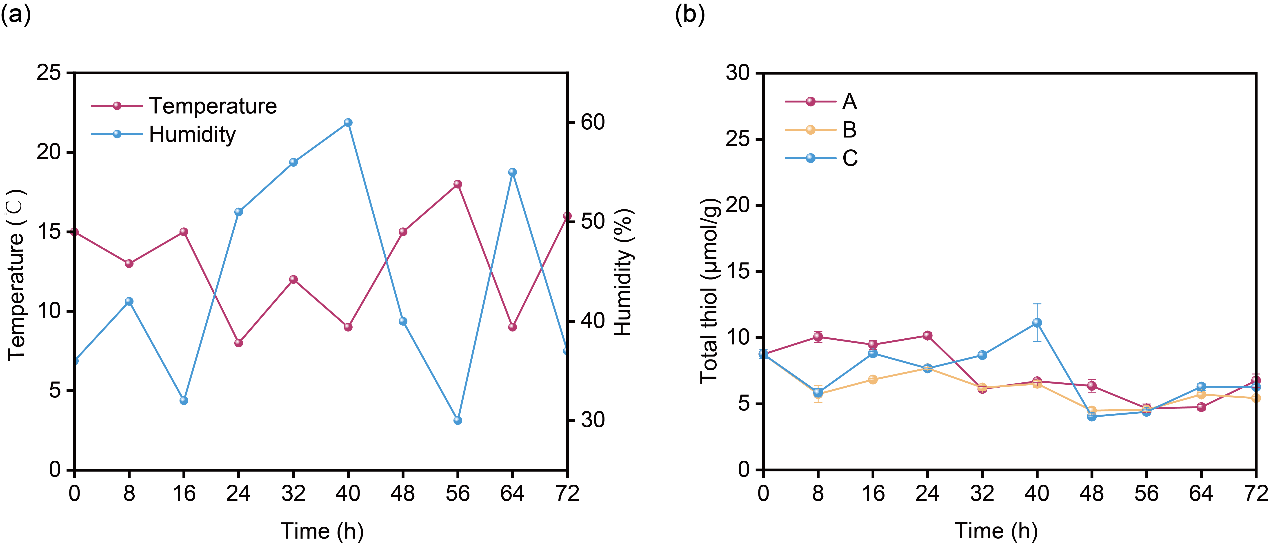


**Fig. S1. Environmental parameters and protein oxidation in grass carp during storage under different packaging conditions.** (a) Temperature and relative humidity profiles during storage. (b) Changes in total sulfhydryl content of muscle proteins under different treatments.


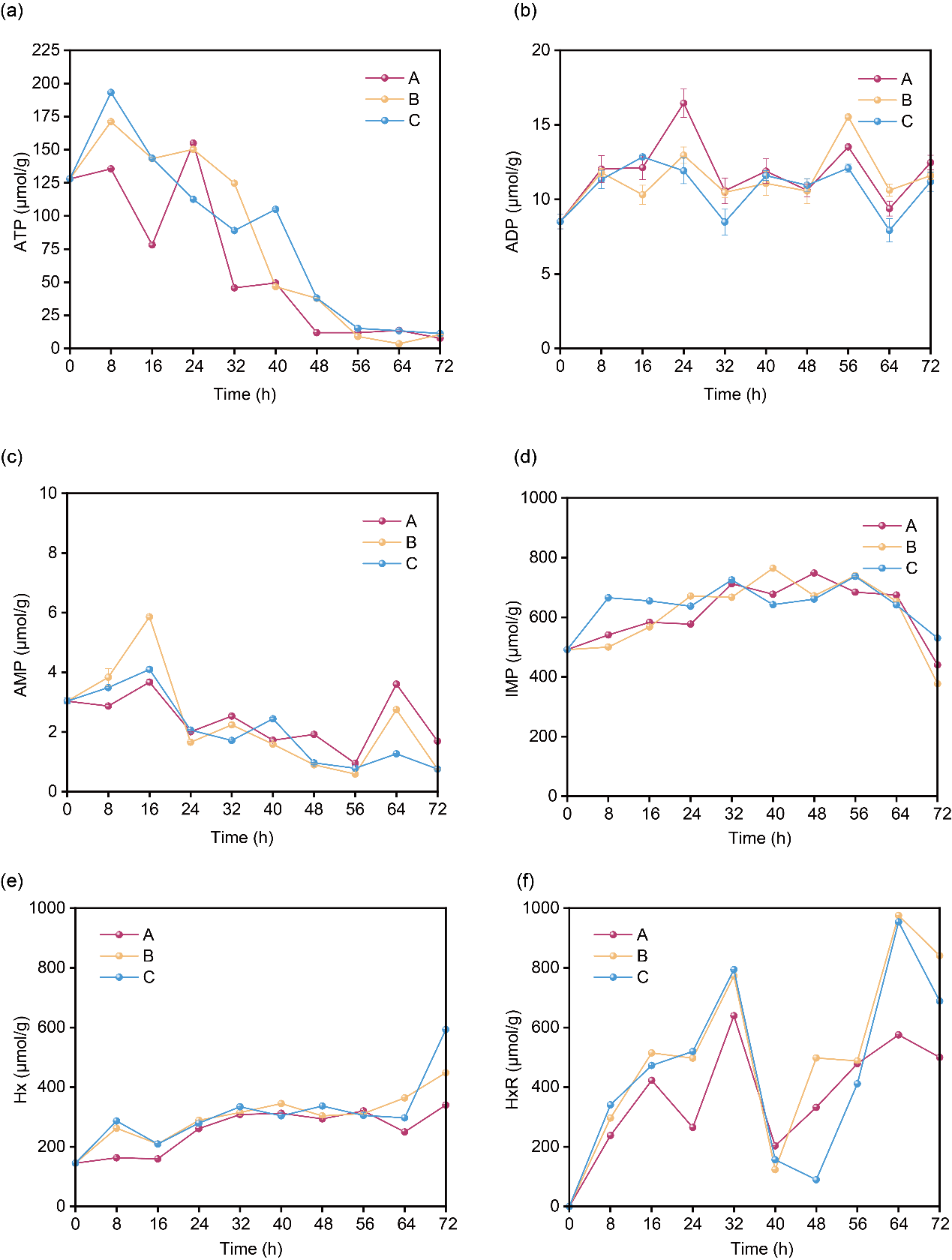


**Fig. S2. Dynamics of ATP and its degradation products in grass carp muscle during storage under different packaging conditions.** (a–f) Changes in ATP, ADP, AMP, IMP, HxR, and Hx contents over storage time across treatments.


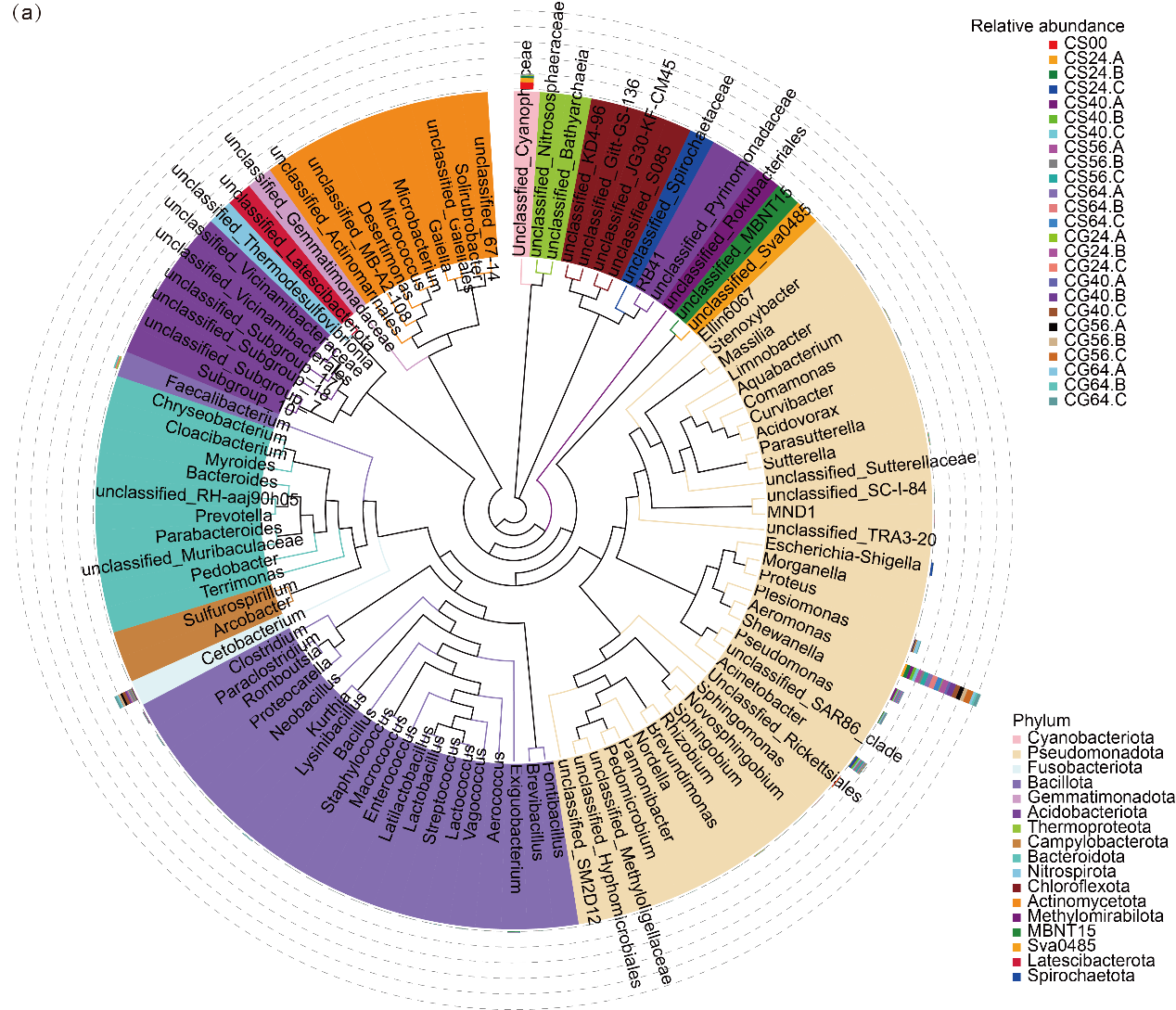


**Fig. S3. Phylogenetic relationships of dominant bacterial genera with abundance profiles across three packaging treatments during storage.**


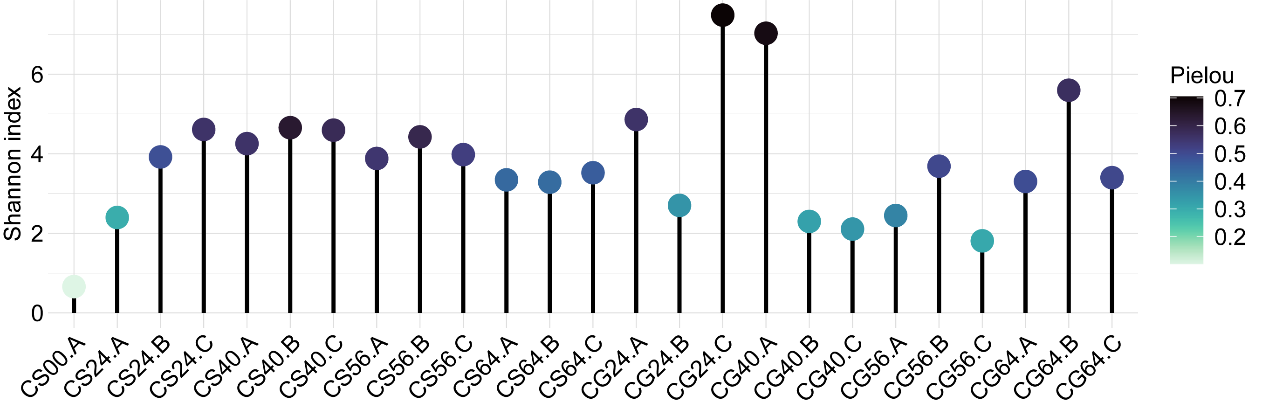


**Fig. S4. Alpha diversity (Shannon and Pielou’s evenness indices) of muscle tissue and gut bacterial communities in grass carp under different packaging conditions.**


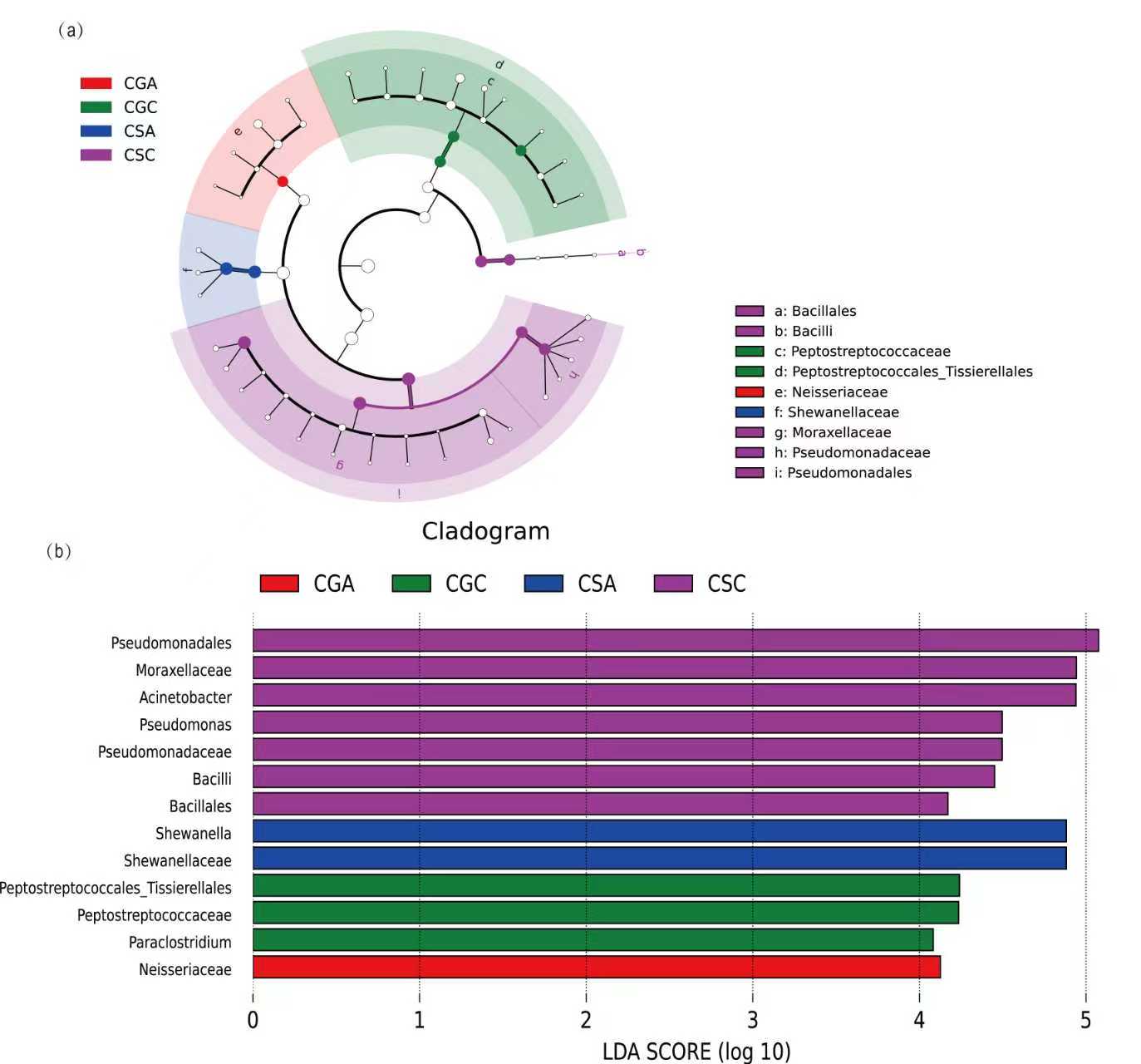


**Fig. S5. LEfSe analysis of differential bacterial taxa in muscle tissue and gut samples of grass carp under different packaging conditions.** (a) Cladogram showing group-specific bacterial biomarkers. (b) LDA scores of significantly enriched taxa. No differential bacterial taxa were identified in group B of muscle tissue and gut samples. CGA, group A of gut samples; CGB, group B of gut samples; CGC, group C of gut samples; CSA, group A of muscle samples; CSB, group B of muscle samples; CSC, group C of muscle samples.
